# Modulation of Endoplasmic Reticulum Stress in Experimental Anti-Cancer Therapy

**DOI:** 10.3390/ijms26136407

**Published:** 2025-07-03

**Authors:** Natalia Ivanovna Agalakova

**Affiliations:** Sechenov Institute of Evolutionary Physiology and Biochemistry, Russian Academy of Sciences, 44 Thorez Avenue, Saint-Petersburg 194223, Russia; nagalak@mail.ru

**Keywords:** cancer cells, endoplasmic reticulum stress, unfolded protein response, SERCA inhibitors, PERK inhibitors, IRE1α inhibitors, ATF6 inhibitors, GRP78 inhibitors

## Abstract

The growth of tumor cells is accompanied by an increased rate of endoplasmic reticulum stress (ERS), the accumulation of misfolded proteins, and the activation of a network of adaptive signaling pathways known as the unfolded protein response (UPR). Although the UPR is an adaptive reaction aiming to restore ER proteostasis, prolonged and severe ERS leads to cell death. Taking into account that the components of the ERS/UPR machinery in cancers of different types can be overexpressed or downregulated, both the induction of excessive ERS and suppression of UPR have been proposed as therapeutic strategies to sensitize cells to conventional chemotherapy. This narrative review presents a several examples of using natural and synthetic compounds that can either induce persistent ERS by selectively blocking ER Ca^2+^ pumps (SERCA) to disrupt ER Ca^2+^ homeostasis, or altering the activity of UPR chaperones and sensors (GRP78, PERK, IRE1α, and ATF6) to impair protein degradation signaling. The molecular alterations induced by miscellaneous inhibitors of ERS/UPR effectors are described as well. These agents showed promising therapeutic effects as a part of combination therapy in preclinical experimental settings; however, the number of clinical trials is still limited, while their results are inconsistent. Multiple side effects, high toxicity to normal cells, or poor bioavailability also hampers their clinical application. Since the pharmacological modulation of ERS/UPR is a valuable approach to sensitize cancer cells to standard chemotherapy, the search for more selective agents with better stability and low toxicity, as well as the development of more efficient delivery systems that can increase their therapeutic specificity, are highly required goals for future studies.

## 1. Introduction

The progression of malignancies of different origin shares a common pathogenesis, including an increased rate of endoplasmic reticulum stress (ERS) caused by genetic mutations and genomic rearrangements, the accumulation of misfolded proteins, and the subsequent activation of multiple signaling pathways that are together known as the unfolded protein response (UPR) [1,2]. A sustained UPR plays an important role in supporting cancer growth, survival, invasion, and metastasis. The ability of tumor cells to rapidly adapt to stress conditions such as limited oxygen and nutrient supplies by activating/deactivating the UPR determines either the resistance to chemotherapy/radiotherapy or a positive therapeutic response [1,2,3,4]. Additionally, the defense mechanisms induced by ERS are implicated in the processes occurring in the tumor microenvironment, such as angiogenesis [5]. On the other hand, persistent ERS promotes cell death, and its induction via different mechanisms is believed to be a promising approach in anti-tumor therapy [3,6]. Moreover, since different components of ERS/UPR pathways in cancers are upregulated or repressed, their expression profiles might be used for diagnostics, determining the prognosis of disease progression, choosing therapeutic schemes, and assessing the response to chemotherapeutics.

**Figure 1:**
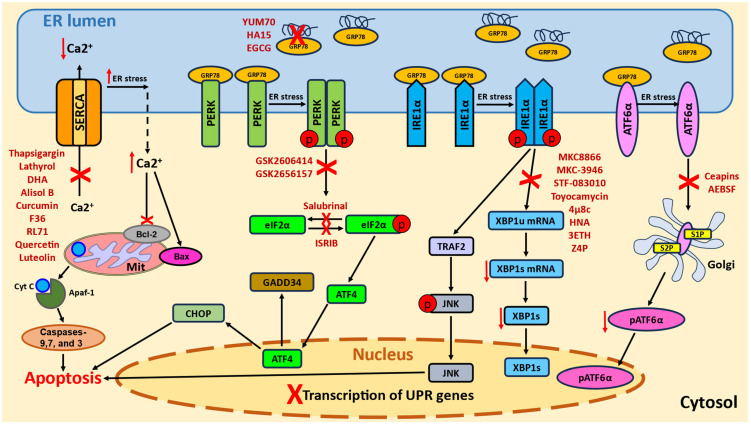
Schematic diagram illustrating the strategies to modulate endoplasmic reticulum (ER) stress in cancer cells. Shown are the unfolded protein response (UPR) molecular network and the compounds used to induce ER stress or disrupt UPR signaling. SERCA—sarcoplasmic/endoplasmic reticulum Ca^2+^-ATPase (pump). SERCA transports Ca^2+^ into the ER lumen, thus maintaining a high ER Ca^2+^ content but a low cytoplasmic Ca^2+^ content. Thapsigargin, lathyrol, DHA, alisol B, curcumin, F36, RL71, quercetin, and luteolin—SERCA inhibitors used to induce ER stress. Targeting SERCA causes ER Ca^2+^ depletion and ER stress, but increases the cytosolic Ca^2+^ level followed by mitochondrial Ca^2+^ overload and dysfunction leading to apoptosis. PERK, IRE1α, and ATF6α are ER stress sensors. Upon the accumulation of unfolded/aggregated proteins in the ER lumen (ER stress) they are released from the chaperone GRP78 and trigger multiple signaling cascades involving downstream transcription factors (ATF4, XBP1s, and pATF6α) and associated proteins, while GRP78 interacts with unfolded proteins. In concert, these mechanisms are able to resolve the misfolded ER protein load and restore protein homeostasis (UPR). GSK2606414 and GSK2656157—PERK inhibitors; salubrinal and ISRIB—eIF2α inhibitors; MKC8866, MKC-3946, STF-083010, toyocamycin, 4µ8c, HNA, 3ETH, and Z4P—IRE1α/XBP1 inhibitors; ceapins and AEBSF—ATF6α blockers; YUM70, HA15, and EGCG—GRP78 blockers. The inhibition of ER sensors or the chaperone GRP78 decreases the ability of cells to cope with ER stress and suppresses the transcription of UPR genes, thus inducing persistent ER stress and promoting pro-apoptotic signaling leading to cell death. X—inhibition, ↑—increase, ↓—downregulation, P—phosphorylation.

The development of novel anti-cancer drugs is extremely time-consuming and cost-ineffective; therefore, the modulation of ERS/UPR for cancer treatment is an attractive alternative in clinical oncology to sensitize the tumor cells to conventional cytotoxins. In this rapidly growing field of research, not only the development of new, selective agents targeting ERS/UPR effectors but also the repurposing of long known herbal or synthetic drugs in combination therapy have emerged [3,6,7,8]. Pro-apoptotic ERS-dependent anti-tumor activity could be achieved either by suppressing the adaptive UPR or by exaggerating ERS [4]. This narrative review highlights recent experimental pharmacological strategies involving a few natural and synthetic blockers used to induce ERS or disrupt UPR signaling. It focuses on miscellaneous compounds either suppressing ER Ca^2+^ pumps (SERCA) to compromise ER Ca^2+^ homeostasis or directly targeting UPR sensors to impair the protein degradation machinery (Figure 1).

## 2. Endoplasmic Reticulum Stress and the Unfolded Protein Response

The endoplasmic reticulum (ER) is a continuous interconnected membrane network consisting of rough ER serving for the synthesis, folding, maturation, and transport of proteins, and smooth ER responsible for the biosynthesis of lipids. Moreover, the ER is a cellular Ca^2+^ store. Such multiple functions make the ER an important contributor to the maintenance and dynamic regulation of cellular proteostasis, lipid metabolism, redox balance, and Ca^2+^ homeostasis and signaling [9,10]. The disruption of the ER folding capacity accompanied by the accumulation of unfolded/aggregated proteins leads to a condition known as ER stress (ERS) [1,2,10]. ERS can be induced by various physiological and pathological stimuli such as metabolic alterations, oxidative stress, hypoxia, chronic inflammation, senescence, and a Ca^2+^ imbalance. To clear the misfolded proteins and enhance the ER folding capacity, cells evolved a protective mechanism—unfolded protein response (UPR)—that includes several transcriptional and translational modifications serving to attenuate protein synthesis and restore ER homeostasis [10,11,12].

The UPR is directed by three transmembrane ER sensors: PERK (PKR-like endoplasmic reticulum kinase), IRE1 (inositol-requiring enzyme 1α) and ATF6 (activating transcription factor 6) [1,2,12,13]. Under healthy conditions, these proteins are inactive due to binding of their luminal domains to the chaperone GRP78 (glucose-regulated protein 78, also known as binding immunoglobulin protein, BiP, or human heat shock protein 5, HSPA5). In response to an increased ER load with misfolded/aggregated proteins, ER sensors dissociate from GRP78 and activate downstream signaling pathways, while GRP78 interacts with unfolded proteins. PERK undergoes dimerization and autophosphorylation allowing it to phosphorylate eIF2α (eukaryotic initiation factor 2α), which inhibits translation and stops the global synthesis of new proteins, a key mechanism of the UPR [14,15]. Another PERK target is ATF4 (activating transcription factor 4), which controls the transcription of adaptive UPR genes and the apoptotic/autophagy genes CHOP (C/EBP protein homolog) and GADD34 (growth arrest and DNA damage-inducible protein 34) [16]. Similar dimerization and autophosphorylation of IRE1 facilitates its RNase activity and triggers the splicing of XBP1 (X-Box binding protein 1) [17,18]. sXBP1 functions as a transcription factor that is able to restore ER homeostasis by enhancing the expression of genes encoding ER chaperones and effectors of the ERAD (ER-associated degradation) system. Another function of IRE1 is the selective destruction of mRNAs and miRNAs encoding compromised ER proteins through a mechanism known as RIDD (regulated IRE1-dependent decay) [19]. The third branch of the UPR includes conformational changes in ATF6, which drives its trafficking to the Golgi apparatus for proteolytic cleavage by serine protease site-1 (S1P) and metalloprotease site-2 (S2P) into the active transcription fragment pATF6α [2,13]. This factor further translocates to the nucleus and stimulates the transcription of XBP1, UPR genes including GRP78, and genes encoding ERAD proteins. In concert, UPR pathways can reduce ERS or restore the ER protein balance by repairing the moderately misfolded proteins but degrading terminally damaged ones. However, if ERS is excessive or persistent, the cells are unable to restore ER homeostasis, and the UPR switches from pro-survival to pro-death signaling [13,20,21]. The effectors connecting the UPR with apoptosis and autophagy include CHOP; JNK (c-Jun N-terminal kinase); the pro-apoptotic Bcl-2 family protein NOXA; caspase-4, which is selectively stimulated by disturbed ER homeostasis; and caspase-12, while the cell destiny depends on the ratio between the activities of these molecules.

## 3. ERS in Cancer Cells

### 3.1. Ca^2+^ Homeostasis, SERCA, and Cancer

The compartmentalization and precise control of the Ca^2+^ distribution in the cells, with a high Ca^2+^ concentration in the ER (100–800 µM) and low cytoplasmic Ca^2+^ levels (50–100 nM), are the phenomena enabling the regulation of various physiological processes, such as gene transcription, cell differentiation, growth, metabolism, migration, survival, or death [22,23]. Strict regulation of the ER Ca^2+^ content is necessary for proper protein synthesis, folding, and maturation. Even a partial ER Ca^2+^ loss enhances the accumulation of unfolded proteins and the UPR with the stimulation of PERK and IREα, while ER Ca^2+^ replenishing leads to complete IRE1α and PERK dephosphorylation [24]. However, a cellular Ca^2+^ imbalance due to altered activities of plasma membrane and ER membrane Ca^2+^ channels and pumps are involved in cancer spread and chemoresistance [23,25,26,27].

SERCA (sarcoplasmic/endoplasmic reticulum Ca^2+^-ATPase) is a P-type transporter located on ER membranes and existing as a few isoforms (1a, 1b, 2a, 2b, and 3). It is responsible for replenishing the ER Ca^2+^ stores by transporting Ca^2+^ from the cytoplasm against its concentration gradient [22,28,29]. In concert with plasma membrane and mitochondrial Ca^2+^ pumps and channels, it plays a crucial role in maintaining cellular Ca^2+^ homeostasis. However, aberrant SERCA activity results in the depletion or overload of ER Ca^2+^ stores and triggers ERS. Since luminal Ca^2+^ binds to ER chaperones like GRP78, calnexin, or calreticulin, an ER Ca^2+^ loss changes their functioning [22,28].

The overexpression, mutations, or loss of different SERCA isoforms are implicated in various malignancies (Table 1). The upregulation of SERCA1-encoding genes is linked to the reduced survival of patients with breast [30] and colorectal [31] cancers. Increased SERCA2 expression in the tissues of patients with colorectal adenoma and carcinoma positively correlates with the tumor grade and metastasis [32,33,34]. The downregulation of SERCA3 determines the invasion and metastasis of gastric carcinomas [35,36], gliomas, and glioblastomas [37], although it is not linked to the survival of patients with colorectal carcinoma [38]. Decreased SERCA3 expression is observed in choroid plexus tumors [39], while strong SERCA3 immunopositivity is found in gastrointestinal tumors [40]. Both SERCA3 loss [41] and overexpression [37] are found in breast carcinomas of various types.

In spite of such variable expression in tumors of different types, SERCAs attracted great attention as targets for anti-cancer therapy [25,26,27,28,29]. Since SERCA overexpression is a predominant phenomenon, the therapeutic strategies are mainly focused on blocking its activity. SERCA suppression induces aberrant ERS signaling and the depletion of luminal Ca^2+^, while concomitant excessive cytoplasmic Ca^2+^ levels cause its entrance into the mitochondrial matrix, mitochondrial Ca^2+^ overload, and depolarization of inner mitochondrial membranes. The following events include the activation of pro-apoptotic Bcl-2 family proteins (Bax and Bak), Cytochrome C release, and the formation of the apoptosome Apaf-1. High cytosolic Ca^2+^ levels also stimulate the protease calpain, which cleaves ER-associated procaspase-12, leading to the activation of caspases-9 and -3, and eventually cell death [2,22].

### 3.2. UPR Components and Cancer

Abnormal activities of all UPR effectors (PERK, IRE1α, and ATF6) have been shown to be implicated in the survival, metastasis, angiogenesis, and chemoresistance of glioblastoma [42], pancreatic [43,44], lung [45], colon/colorectal [46,47], and urological [48] cancers at different stages of the disease. The overexpression, downregulation, phosphorylation, and mutation of several eIF subunits (eIFs) in malignancies of various origins have been reported as well [49,50]. The IRE1α/XBP1 branch is implicated in the development of a variety of hematologic [51,52] and pancreatic [53] cancers. GRP78 also emerges as a central player in the pathogenesis of and chemoresistance in many cancers [54,55].

***PERK/eIFα***. Increased PERK levels in the tumor tissues are associated with a poor prognosis for patients with prostate cancer [56], pancreatic duct adenocarcinoma (PDAC) [57], kidney renal papillary cell carcinoma, gliomas, breast carcinoma, and thyroid carcinoma [58], but determined a favorable prognosis for head and neck squamous cell carcinoma (HNSCC) [58] (Table 2). In contrast, it was downregulated in Hodgkin’s lymphoma, T-cell lymphomas, and gastrointestinal stromal tumors [58].

eIF2α overexpression was found in tumors originating from melanocytes and the colonic epithelium [59], in aggressive thyroid cancers [60], Hodgkin’s lymphoma [61], and the tumor tissues and blood plasma of breast cancer patients [62,63]. Sustained eIF2α phosphorylation was linked with a poor prognosis, higher risk of metastasis, and lower disease-free survival of patients with prostate cancer [56,64], PDAC [57], pancreatic adenocarcinoma (PAAD) [65], hepatocellular carcinoma (HCC) [66], and brain tumors [67]. On the other hand, eIF2α hyperphosphorylation correlated with better disease-free survival and favorable clinical outcomes for patients with stomach, colon and sigma-rectum carcinomas [68], non-small cell lung cancer (NSCLC) [69], and triple-negative breast cancer (TNBC) [70].

***IRE1α/XBP1***. The upregulation of IRE1α was found to be an independent factor predicting a higher recurrence of prostate cancer [56,71]. In contrast, in lung adenocarcinoma, IRE1α overexpression was associated with a favorable prognosis [72].

XBP1 overexpression and splicing (sXBP1) was reported in the tumor tissues of patients with HCC [73], breast adenocarcinoma [74], melanoma [75], and glioblastoma [76]. XBP1 upregulation predicted a poor response to therapy and shorter survival of patients with aggressive diffuse large B-cell and plasmablastic lymphomas [77], acute lymphoblastic leukemia (ALL) [78], aggressive luminal B and ER+ breast cancers [79,80], and prostate cancer [81]. On the other hand, increased levels of mature and spliced XBP1 forms were associated with better overall survival of patients with acute myeloid leukemia (AML) [82,83]. Moderate sXBP1 expression sensitized the cells to cytotoxic treatments, while sustained XBP1 activation induced apoptosis [84]. However, in pancreatic cancer, XBP1 upregulation was not associated with recurrence and overall survival [85].

***ATF6***. Increased levels of the ATF6 mRNA and its transcriptionally active nuclear product pATF6α were observed in the tissues of patients with moderately to poorly differentiated HCC [73] and osteosarcoma [86]. ATF6 overexpression was associated with chemoresistance and poor clinical outcomes of ovarian cancer [87], gastric cancer [88], pancreatic cancer [85], oral squamous cell carcinoma [89], and HNSCC [90]. However, high ATF6 level did not correlate with the overall survival of patients with biliopancreatic carcinoma [91], prostate [56], and colon [92] cancers, although it was a predictor of a shorter recurrence time.

***GRP78***. High expression of GRP78 was found in the tissue lesions and bone marrow of patients with breast cancer [74,93,94]. Abnormally elevated levels of GRP78 correlated with stronger aggressiveness, lower overall survival, increased invasion and metastasis, and low sensitivity to anti-cancer agents in patients with HCC [73,95,96], gastric cancer [97], gliomas [98], prostate cancer [99], PDAC [100], lung cancer [101,102], and HNSCC [103]. In contrast, high GRP78 expression in the tissues of patients with colorectal cancer was associated with improved survival after surgery [104].

**Table 2:** Examples of the aberrant expression of UPR effectors in human cancer tissues.

Effector	Expression Profile	Cancer Type	Clinical Outcome	References
**PERK**	Overexpression	Prostate cancer	Poor prognosis	[56]
		Pancreatic duct adenocarcinoma		[57]
		Kidney renal papillary cell carcinoma		[58]
		Brain glioma		[58]
		Breast carcinoma		[58]
		Thyroid carcinoma		[58]
		Head and neck squamous cell carcinoma	Favorable prognosis	[58]
**eIF2α**	Overexpression, Phosphorylation	Melanoma, colonic adenoma and adenocarcinoma	-	[59]
		Aggressive thyroid carcinoma and papillary carcinoma		[60]
		Hodgkin’s lymphoma		[61]
		Prostate cancer	Poor prognosis Lower overall and disease-free survival Metastasis Chemoresistance	[56]
		Pancreatic duct adenocarcinoma		[57]
		Pancreatic adenocarcinoma		[65]
		Hepatocellular carcinoma		[66]
		Brain meningioma, astrocytoma and oligodendroglial tumors		[67]
	Overexpression, Phosphorylation	Stomach, colon, sigma-rectum carcinoma	Longer survival Better disease-free survival	[68]
		Non-small cell lung cancer		[69]
		Triple-negative breast cancer		[70]
**IRE1** **α**	Overexpression	Prostate cancer	Higher recurrence	[56,71]
		Lung adenocarcinoma	Favorable prognosis	[72]
**XBP1/sXBP1**	Overexpression Splicing	Hepatocellular carcinoma	-	[73]
		Breast adenocarcinoma		[74]
		Melanoma		[75]
		Glioblastoma		[76]
		Diffuse large B-cell lymphoma	Shorter overall and disease-free survival Poor response to therapy	[77]
		Acute lymphoblastic leukemia		[78]
		Aggressive luminal B breast cancer		[79]
		ER+ breast cancer		[80]
		Prostate cancer		[81]
		Acute myeloid leukemia	Better disease-free and overall survival Lower relapse rate	[82,83]
		Pancreatic cancer	No correlation with survival	[85]
**ATF6**	Overexpression	Hepatocellular carcinoma	Poor prognosis Chemoresistance, lower overall survival	[73]
		Osteosarcoma		[86]
		Ovarian cancer		[87]
		Gastric cancer		[88]
		Pancreatic cancer		[85]
		Oral squamous cell carcinoma		[89]
		Head and neck squamous carcinoma		[90]
		Biliopancreatic carcinoma	No correlation with survival	[91]
		Prostate cancer		[56]
		Colon cancer		[92]
**GRP78**	Overexpression	Breast adenocarcinoma	-	[74,93,94]
		Hepatocellular carcinoma	Poor prognosis Lower overall survival Invasion Metastasis Chemoresistance	[73,95,96]
		Gastric cancer		[97]
		Gliomas		[98]
		Prostate cancer		[99]
		Pancreatic duct adenocarcinoma		[100]
		Lung cancer		[101,102]
		Head and neck squamous cell carcinomas		[103]
		Colorectal cancer	Improved survival	[104]

Comments: - not analyzed.

Such diverse findings encouraged using both the induction of ERS and inhibition of UPR effectors as therapeutic approaches. Strong anti-tumor activity caused by chronic ERS followed by apoptosis was achieved in primary and stable leukemic cell lines [51] and ovarian cancer [105]. A variety of natural, synthetic, and semisynthetic compounds either activating or suppressing IRE1α, PERK, ATF6, or GRP78 has been tested for the treatment of breast cancer [7,106], thyroid cancer [8], and glioma/glioblastoma [107,108]. A few agents that stimulate the UPR entered clinical trials for pancreatic cancers [44]. Suppression of the adaptive UPR makes tumor cells more susceptible to chemotherapy as well [109,110].

## 4. SERCA as a Target for Cancer Treatment

To date, a few chemically and functionally diverse SERCA inhibitors of natural (terpenoids, polyphenols, and flavonoids) or synthetic origin have been developed and examined [29,111,112].

### 4.1. Terpenoids

***Thapsigargin (TG)***, a terpenoid with a long history of use in traditional medicine, is a highly selective irreversible SERCA blocker that prevents Ca^2+^ transport from the cytosol to ER, thus depleting the ER Ca^2+^ stores but increasing the cytosolic Ca^2+^ content [113,114]. TG is a SERCA inhibitor that has been probably most extensively tested for anti-cancer therapy [115,116]. It effectively suppressed the growth of cultured prostate, renal, bladder, colon, thyroid, and breast cancer cells [117]. In conventional (MCF7) and metastatic (MDA-MB-231) breast cancer cells, TG increased the generation of ROS, activated PARP and caspases-8, -9 and -3 [118,119], reduced the mRNA and protein levels of Ca^2+^-binding protein S100A4 [120], and induced the contraction and rearrangement of actin cytoskeleton with the phosphorylation of myosin light chain 2 kinase and myosin phosphatase 1 [121] (Table 3). The effects of TG on prostate cancer LNCaP and PC3 cells include the sustained activation of the death receptor DR5, chaperones GRP94 and GRP78, PERK/ATF4/CHOP and IRE1/XBP1/JNK cascades, and cleavage of PARP and caspase-8 downstream of MAP1LC3B [118,122]. In patient-derived stem cell-enriched glioblastoma cultures, TG impaired the ability of cells to form neurospheres by decreasing the expression of the stem cell transcription factor SOX2 but stimulating the UPR and apoptosis [123]. The disturbance in ER Ca^2+^ homeostasis in neuroblastoma SH-SY5Y cells was accompanied by the activation of caspase-4 [124]. Mitochondrial apoptosis and ERS through JNK signaling with the upregulation of the UPR and autophagy are implicated in TG’s effects on adrenocortical carcinoma SW-13 and NCI-H295R cells and mouse ACC xenograft models [125]. Increased expression of CHOP, DR5, and Bid and the cleavage of PARP and caspases-3 and 8 were observed in TG-exposed liposarcoma SW872 cells [126].

In combination therapy, TG exerted additive or synergistic effects with a variety of cytotoxic or anti-proliferative agents. It enhanced the responses of metastatic and cisplatin-resistant breast cancer cells to cisplatin [127,128]. Although not effective as a single agent, TG increased the sensitivity of patient-derived papillary thyroid cancer (PTC) YUMC cells and mouse YUMC xenograft models to paclitaxel, sorafenib and lenvatinib [129,130]. In oral cancer CAL 27 and Ca 9–22 cells, TG potentiated the effect of the Ca^2+^ channel blocker manoalide, a sesterterpenoid isolated from the marine sponge *Luffariella variabilis* [131]. In breast cancer MCF7 and MDA-MB-231 cells, it synergized with the anti-proliferative effect of nodakenin, a bioactive coumarin glycoside from *Angelica gigas* [132], and physapruin A, a withanolide from the plant *Physalis peruviana* [119].

***Thapsigargin analogues***. In spite of strong ERS induction and the promising results obtained for cultured cancer cells and animal xenograft models, clinical TG application is not possible due to the unacceptably high toxicity to normal cells [113,117]. As a result, a series of TG water-soluble cell-impermeable prodrugs conjugated to peptides that are specifically cleaved by PSA (prostate-specific antigen) or PSMA (prostate-specific membrane antigen) have been designed [133,134]. Some of them suppressed the viability of prostate cancer cells and the growth of LNCaP prostate xenografts in mice without substantial systemic toxicity [117,118]. One of the PSMA-hydrolyzed prodrugs, mipsagargin (G-202), was accepted for the therapy of multiple advanced solid tumors (prostate cancer, glioblastoma, and hepatocellular carcinoma) in clinical trials [133,134]. However, although G-202 was relatively well tolerated and some patients previously heavily treated with chemotherapy or radiotherapy demonstrated longer disease stabilization, objective clinical responses were not found [135,136]. A recently completed trial that examined the efficacy and safety of G-202 for the treatment of glioblastoma did not show any clear clinical outcomes as well [137]. Due to reported side-effects and other reasons, G-202 was not registered [134], and its role as an anti-cancer agent remains uncertain.

***Other terpenoids***. Lathyrol is a natural terpenoid extracted from the seeds of the Asian plant *Euphorbiae lathyrism.* In lung tumor A549 and H460 cells, it triggered apoptosis and G2/M cell cycle arrest accompanied by a cytoplasmic Ca^2+^ elevation, ROS production, and the upregulation of apoptotic and ERS-related proteins [138]. It also greatly suppressed the growth of subcutaneous H460 tumors in mice without significant effects on the liver, heart, lungs, and kidneys [138]. The downregulation of Ki67, Bcl-2, metalloproteinases, and p-Akt, but overexpression of Bax and cleaved caspases underlie the impaired proliferation and increased apoptosis of renal cell carcinoma exposed to lathyrol [139]. Dihydroartemisinin (DHA), a derivative of natural artemisinin that was used to treat malaria, exerted synergistic anti-proliferative, anti-metastatic, and pro-apoptotic effects in combination with a series of chemotherapy drugs. However, although it effectively inhibits SERCA activity and increases ERS, its clinical application is limited due to low solubility and bioavailability [140]. Another group of natural compounds showing cytotoxic and antimetastatic activity in cancer cells of different origins, including those resistant to chemotherapy, are triterpenes isolated from *Alisma* species plants, including alisol B [141].

### 4.2. Curcumin and Its Analogues

***Curcumin***, a polyphenol isolated from the plant *Curcuma longa*, is a potent allosteric SERCA blocker that induces conformational changes in its molecule, thus preventing ATP binding [142,143]. This compound is well known from traditional medicine and was tested as a part of combination therapy for breast, oral, lung, prostate, hematological, and brain cancers [144]. The cellular effects of curcumin include the inhibition of proteins of the Wnt/β-catenin, PI3K/Akt, EGFR and NF-kB signaling pathways; cell cycle arrest; ROS production; and changes in miRNA expression [145,146]. It interferes with miscellaneous transcription factors, growth factors, inflammatory cytokines, apoptotic proteins, protein kinases, receptors, and cell survival proteins [144].

A causative link between the anti-neoplastic effects of curcumin and the disruption of Ca^2+^ homeostasis was shown in ovarian cancer SKOV3, MDAH2774, and PA1 cells [147]. In human liposarcoma SW872 cells and SW872 xenografts, curcumin enhanced eIF2α phosphorylation and increased the expression of ATF4, CHOP, DR5 receptor, and its effectors (caspases-8 and -3, and Bid) [126] (Table 3). The effect of curcumin on glioma LN229 and U87 cells and LN229 xenografts was linked to the suppression of ERK1/2 phosphorylation, metalloproteinases, and cell cycle proteins [148]. By activating both apoptosis and ERS, it increased the sensitivity of cisplatin-resistant NSCLC A549 and H1299 cells to cisplatin [149]. In a combination therapy, curcumin derivatives synergistically enhanced the effect of temozolomide on glioblastoma U87 and LN18 cells [150]. A recent meta-analysis has confirmed its potency to reduce the volume of glioma xenografts in animals [151].

However, in spite of the promising in vitro effects, the effectiveness of curcumin in clinical trials is still not clear due to the high heterogeneity of the obtained results [152]. Thus, the therapy of breast cancer with docetaxel, paclitaxel, gemcitabine, or radiotherapy in combination with curcumin improved the objective response rate and reduced the severity of side effects such as dermatitis [146]. On the other hand, the addition of curcumin to first- or second-line docetaxel treatment of advanced metastatic breast cancer was not effective, with only an insignificant tendency to longer 12-month progression-free survival [153]. The causes of such inconsistency are probably the low chemical stability of curcumin, its limited water solubility, poor bioavailability after oral intake, and rapid elimination from an organism. The development of more effective ways of delivering curcumin to cancer cells would increase its therapeutic specificity in clinical applications.

***Curcumin analogues***. To enhance the therapeutic potential of curcumin, its derivatives with better bioavailability and stability that might improve anti-cancer effectiveness have been developed [154]. The curcumin analogue F36 exhibited more potency in suppressing SERCA2 and inducing ERS-associated apoptosis in human colon adenocarcinoma epithelial SW480 cells [33]. Similar effects were observed on SW480 cells and mice with xenografts that were treated with the curcumin analog RL71 [155].

### 4.3. Flavonoids

***Quercetin***. Among the miscellaneous natural SERCA blockers, flavonoids emerge as valuable agents for preventive or therapeutic anti-cancer purposes [29,111]. Quercetin and its derivatives are able to induce conformational changes in the SERCA molecule [156]. They exert anti-proliferative and anti-apoptotic activities in cancers of different origins by modulating the activities of components of the PI3K/Akt/mTOR, MAPK/ERK, JAK/STAT, and NF-κB signaling pathways [157,158]. Thus, in hepatocellular carcinoma, quercetin promoted apoptosis by inhibiting PI3K/Akt/mTOR cascade proteins and P4HA2 (proline 4-hydroxylase II), an enzyme involved in the biosynthesis of collagen [159]. In acute myeloid leukemia HL-60 cells, it induced both apoptosis and autophagy through the CaMKKβ/-dependent pathway, which included AMPK phosphorylation and the suppression of p-mTOR [160]. The pro-apoptotic effect of quercetin on cutaneous melanoma A375 cells was associated with the activation of GPER (G-protein coupled estrogen receptor) and enhanced expression of p-ERK and c-Myc [161]. A derivative of quercetin, dihydroquercetin, has been tested as anti-cancer agent as well [162].

***Luteolin***. Luteolin, another natural flavonoid with anti-inflammatory, antioxidant, and anti-cancer properties, induced the same intracellular changes as quercetin in melanoma A375 cells [161]. The anti-proliferative and pro-apoptotic effects of luteolin on non-small cell lung cancer H1299 and A549 cells were associated with the downregulation of the components of the WDR72 (WD repeat domain 72), Akt and EMT (epithelial–mesenchymal transition) axis necessary for protein interactions, cytoskeletal regulation, and cell migration [163]. It also reduced the formation of subcutaneous H1299 tumors in mice without evident damage to major organs [164]. In bladder cancer EJ138 cells, luteolin increased the expression levels of genes encoding apoptosis and autophagy-related proteins [163]. The anti-tumor activity of luteolin in cultured diffuse large B-cell lymphoma U2932 and OCI-LY10 cells and mouse subcutaneous U2932 xenograft models included the suppression of JAK2/STAT3 phosphorylation alongside with changes in the expression of apoptotic proteins [165].

However, as other natural compounds, flavonoids have limited bioavailability. To enhance their potency and chemosensitivity, a variety of more efficient delivery systems, including liposomes and nanoparticles, is currently under development [166,167,168,169].

### 4.4. Other SERCA Inhibitors

The attempts to design agents with SERCA-targeted, ERS-inducible properties but low toxicity continues. A few compounds of different chemical natures (named by the authors as compounds 7, 13, 40, 42, etc.) [129,130], as well as the small inhibitors CKP1 and CKP2 with high SERCA1 binding affinity but lower cardiac toxicity than TG [170], augmented apoptosis in patient-derived PTC YUMC cells, including chemotherapy-resistant lines, in the presence of paclitaxel, sorafenib or lenvatinib, and reduced the growth of mouse xenograft originated from YUMC stem-like cells. Four small molecules were lately proposed as potential SERCA1 inhibitors for colorectal carcinoma cells [31]. The natural arylnaphthalide lignan diphyllin, a blocker of vacuolar H^+^-ATPase, was also recently tested as a SERCA2 inhibitor. In NSCLC cells, diphyllin promoted ERS and apoptosis by directly suppressing SERCA2 activity, leading to ER Ca^2+^ depletion, cytoplasmic and mitochondrial Ca^2+^ accumulation, increased ROS production, a decreased mitochondrial membrane potential, and cytochrome C release [171]. Moreover, it exerted synergistic anti-tumor effects in combination with cisplatin in vitro and in vivo. A few diphyllin derivatives with longer half-lives, better bioavailability and metabolic stability more effectively blocked autophagy and induced apoptosis in pancreatic cancer [172] and HNSCC cells and xenografts [173].

**Table 3:** Examples of SERCA inhibitors used for the induction of unresolved ERS in cancer cells.

Cell Type	Treatment	Molecular Changes	Cellular Effects	References
		**Terpenoids**		
Breast cancer MCF7 and MDA-MB-231 cells	TG 6–100 nM 6–48 h	↓ ER Ca^2+^, ↑ROS ↑ Cleaved PARP ↑ Caspases-8, 9, 3	↑ Cells in subG1 phase ↓ Proliferation ↓ Viability, ↑ Apoptosis	[118,119]
Breast cancer cells MDA-MB-231 and MDA-MB-436	TG 2–10 µM 6 or 24 h	↑ Cytoplasmic Ca^2+^ ↑ p-Myosin light chain 2 kinase ↑ p-Myosin phosphatase 1	Actin contraction and rearrangement Changed morphology	[121]
Prostate cancer LNCaP, PC3 cells	TG 30–100 nM 6–48 h	↓ ER Ca^2+^, ↑ GRP94, ↑ GRP78, ↑ ATF4, ↑ cleaved PARP, ↑ CHOP	↓ Proliferation, ↑Death Changed morphology	[118]
Transfected prostate cancer LNCaP cells	TG 100 nM 30–48 h	↑ DR5, ↑ PERK, ↑ ATF4, ↑ CHOP ↑ IRE1, ↑ XBP1, ↑ JNK ↑ Cleaved PARP, ↑ Caspases-3, 8	↑ Cell death	[122]
Patient-derived stem cell-enriched glioblastoma culture	TG 1–10 µM 24–48 h	↑ p-PERK, ↑ ATF4, ↑ CHOP, ↑GRP78, ↑ sXBP1, ↑ ATF6, ↓ SOX2, ↑ Cleaved PARP, ↑ Caspases-3/7	↓ Viability ↓ Neurosphere-forming ability	[123]
Neuroblastoma SH-SY5Y cells	TG 300 nM 30 min or 4 h	↓ ER Ca^2+^, ↑ ROS ↑ Hypodiploid nuclei, ↑ GRP78, ↑ ATF4, ↑ p-PERK, ↑ Caspase-4	↓ Viability	[124]
ACC SW-13 and NCI-H295R cells	TG 0.5–32 µM 48 h	↑ p-JNK/JNK, ↑ PERK, ↑ ATF6, ↑ LC3B, ↑ HSAP, ↑ Bcl-2	↓ Viability, ↑ Apoptosis, ↓ Migration, invasion	[125]
Mice with SW-13 cell xenografts	TG 1 mg/kg 14 days	↑ p-JNK/JNK, ↑ p-ERK/ERK, ↑ p-PERK/PERK, ↑ GRP78, ↑ IRE1	↓ Tumor growth	[125]
Patient-derived PTC YUMC cells resistant to PTX, SOR, and LEN	TG 10–200 µM + PTX, or SOR, or LEN 10–200 µM 40 h	↑ CHOP, ↑p-PERK ↑ Cytochrome c ↑ Cleaved caspase-3	↑ Sensitivity to PTX, SOR or LEN ↓ Viability	[129,130]
Mice with PTC YUMC xenografts resistant to PTX, SOR, and LEN	TG 25 mg/kg PO + PTX 25 mg/kg IP, or SOR 80 mg/kg PO, or LEN 10 mg/kg PO	↑ CHOP	↓ Tumor weight ↑ Sensitivity to PTX, SOR or LEN	[129,130]
Oral cancer CAL 27 and Ca9–22 cells	TG 10 nM + Manoalide 5–10 µM 24 h	↑ Caspase 3/7	↓ Viability, ↑ Autophagy ↑ Sensitivity to manoalide	[131]
Breast cancer MCF7 and MDA-MB-231s cells	TG 3 µM + nodakenin 40 µM 24 h	↑ p-PERK ↑ p-eIF2α ↑ CHOP, ↑ ATF4	↑ Sensitivity to nodakenin ↑ Cell death	[132]
Hepatocellular carcinoma	G-202 40 mg on 1–3 d, or 40 mg on 1 d and 66.8 mg on 2–3 d of 28-d cycle		No complete response No progressive disease Stable disease Partial response	[135,136]
Glioblastoma multiforme	G-202 IV for 3 days of 28-d cycle		No clear conclusions	[137]
Lung cancer A549 and H460 cells	Lathyrol 30–120 µg/mL 24 h or 14 d	↑ Cytosolic Ca^2+^, ↑ GRP78, ↑ PERK ↑ p-eIF2α, ↑ CHOP, ↑ ATF4, ↑ Bax, ↓ Bcl-2, ↑ Caspase-3, ↑ Cyt C	↓ Viability, ↑ Apoptosis	[138]
Mice with H460 cell xenografts	Lathyrol 10–40 mg/kg IP 16 d		↓ Tumor volume and weight	[138]
Renal cell carcinoma 786-O cells	Lathyrol 10–375 µg/mL 24 h	↓ Bcl-2, ↑ Bax, ↓ p-Akt, ↓ MMP2, ↓ MMP9, ↓ Ki67, ↑ Caspase-3, 9	↓ Viability, ↓ Invasion, ↓ Migration, ↑ Apoptosis	[139]
**Curcumin and its analogues**				
Human liposarcoma SW872 cells	Curcumin 5–20 µM 24 or 48 h	↓ Ca^2+^-ATPase activity, ↑ DR5, ↑ Caspase-8, ↑ Caspase-3, ↑ Bid, ↑ PARP, ↑ CHOP, ↑ p-eIF2a, ↑ ATF4	↓ Cell growth ↑ Apoptosis	[126]
SCID mice injected with SW872 cells	Curcumin 100 mg/kg IP 40 d	↑ Caspase-8, ↑ Caspase-3, ↑ Cleaved PARP, ↑ CHOP,	↓ Tumor growth	[126]
Human glioma LN229 and U87 cells	Curcumin 8–32 μM 24–72 h	↓ Cyclin D1, ↓ CDK46/6 ↓ Bcl-2, ↓ Bcl-XL, ↓ MMP-2, ↓ MMP-9, ↓ p-ERK1/2	↓ Proliferation ↓ Migration and invasion	[148]
Mice with subcutaneous LN229 xenografts	Curcumin 60 mg/kg/d 4 w	↓ MMP-2 and MMP-9 ↓ CD147	↓ Tumor growth	[148]
CIS-resistant NSCLC A549 and H1299 cells	Curcumin 2.5 μg/mL + CIS 2 μg/mL 48 h	↑ Cleaved PARP, ↑ Caspase-3 ↑ GRP78, ↑ ATF6, ↑ XBP1 ↑ Caspase-4, ↑ CHOP	↓ Viability ↑ Apoptosis ↑ Sensitivity to CIS	[149]
Glioblastoma U87 and LN18 cells	Curcumin 10 µg/mL + TMZ 200 µM	Alterations in actin network	Cell cycle arrest, ↓ Viability, ↑ Apoptosis, ↑ Sensitivity to TMZ	[150]
Colon carcinoma SW480 cells	F36 1–10 µM 24–72 h	↑ Cleaved PARP, ↑ Caspase-3, ↑ CHOP, ↑ ATF4, ↑ p-eIF2a	↓ Proliferation ↑ Apoptosis	[33]
Advanced metastatic breast cancer	Curcumin 6 g (7 d) + DOC 100 mg/m^2^ every 3 w 6 cycles		No significant difference in the objective response rate and 12-month overall survival	[153]
Colon carcinoma SW480 cells	RL71 0.5–10 µM 24–72 h	↑ GRP78, ↑ ATF4, ↑ CHOP, ↑ cleaved PARP	↓ Viability G2/M cell cycle arrest	[155]
Mice with SW480 xenografts	RL71 1–4 mg/kg 14 d	↑ Cleaved PARP, ↑ CHOP, ↑ cleaved Caspase-3	↓ Tumor growth	[155]
**Flavonoids**				
HCC SNU-449 and Hep-3B cells	Quercetin 6.5–75 µM 24–48 h	↓ p-PI3K, ↓ p-Akt, ↓ p-mTOR, ↓ Bcl-2, ↑ Bax, ↑ cleaved PARP, ↑ cleaved Caspase-3, ↓ P4HA2	↓ Viability, ↑ Apoptosis, ↓ Proliferation, ↓ Colony formation	[159]
AML HL-60 cells	Quercetin 25–100 µM 24–72 h	↑ LC3II/I ↓ Bcl-2 ↑ Bax ↑ p-AMPK ↓ p-mTOR ↑ Caspase-3	↓ Viability, ↑ Apoptosis, ↑ Autophagy, ↓ Colony formation	[160]
Melanoma A375 cells	Quercetin 1–100 µM 24–72 h	↑ p-ERK, ↑ p-Akt, ↑ GPER, ↑ c-Myc	↓ Viability, Cell cycle arrest, Changed morphology, ↑ Apoptosis/necrosis	[161]
NSCLC A549 and H1299 cells	Luteolin 0.1–1000 µM 12–72 h or 50 µM 24 h	↓ WDR72, ↓ Bcl-2, ↑ Caspase-3, ↓ p-Akt, ↓ E-cadherin, ↓ β-catenin, ↓ N-cadherin, ↓ ZEB1	↓ Viability, ↓ Migration and invasion, ↓ Proliferation	[163]
Mice with NSCLC H1299 xenografts	Luteolin 50 mg/kg IP once/2 d 21 d	↓ WDR72 mRNA	↓ Tumor growth	[163]
Bladder cancer EJ138 cells	Luteolin 20–50 µM 24–48 h	↑ P53, ↑ ULK1, ↑ ATG12, ↓ BCL2	↓ Viability, ↑ Apoptosis G2/M phase arrest	[164]
DLBCL U2932 and OCI-LY10 cells	Luteolin 5–20 µM 24 h	↑ Bax, ↓ Bcl-2, ↑ cleaved PARP, ↑ Caspase-3, ↓ p-JAK2, ↓ p-STAT3	↑ Apoptosis	[165]
Mice with U2932 tumors	Luteolin 12.5–50 mg/kg IP 14 d	↑ Bax, ↓ Bcl-2, ↑ cleaved PARP, ↑ cleaved Caspase-3, ↓ p-JAK2, ↓ p-STAT3	↓ Tumor volume and weight	[165]
**Other SERCA inhibitors**				
Patient-derived PTX-, SOR-, or LEN- resistant PTC YUMC cells	Compounds 7, 13, 40, 42 (10–200 µM) + PTX, SOR, LEN 10–200 µM 40 h	↑ p-PERK, ↑ CHOP, ↑ Cytochrome c, ↑ Cleaved caspase-3	↑ Sensitivity to PTX, SOR, or LEN ↓ Viability	[129,130]
Mice with xenografts of PTX-, SOR-, or LEN-sensitive and -resistant YUMC PTC cells	Compounds 7, 13, 40, 42 (25 mg/kg) + PTX 25 mg/kg, or SOR 80 mg/kg, or LEN 10 mg/kg		↓ Tumor weight ↑ Sensitivity to PTX, SOR, or LEN	[129,130]
NSCLC	Diphyllin	↓ Ca^2+^ levels in the ER, ↑ ROS, ↓ MMP, Cytochrome C release	↓ Proliferation, ↓ Migration, ↑ Apoptois, Synergy with CIS	[171]

Abbreviations: ACC—adrenocortical carcinoma, PTC—papillary thyroid cancer, NSCLC—non-small cell lung cancer, HCC—hepatocellular carcinoma, AML—acute myeloid leukemia, DLBCL—diffuse large B-cell lymphoma. PTX—paclitaxel, LEN—lenvatinib, SOR—sorafenib, TMZ—temozolomide, DOC—docetaxel, CIS—cisplatin, PO—per oral, PI—intravenous, ↑—increase, ↓—suppression.

## 5. UPR Modulators

### 5.1. PERK Inhibitors

***GSK2606414*** is a highly potent, synthetic first-generation PERK inhibitor that competitively targets its ATP-binding site [174]. As a single agent, GSK2606414 suppressed the proliferation of PDAC SW1990 cells with a high level of the tumorigenic BZW1 protein (basic leucine zipper and W2 domain-containing protein 1) and the growth of SW1990 xenografts [175] (Table 4). The application of GSK2606414 with the autophagy inhibitor simvastatin sensitizes glioblastoma U87 and U251 cells to temozolomide, although it differentially affected autophagosome formation (LC3β-II/LC3β-I ratio) [176]. The combination of GSK2606414 and irradiation led to sustained ERS followed by apoptosis in glioblastoma cells in vitro, and enhanced the overall survival of mice with orthotopic glioblastoma by preventing tumor recurrence [177]. PERK suppression sensitized multidrug-resistant colorectal cancer S1-M1–80 cells overexpressing the cancer resistance protein ABCG2 to doxorubicin and mitoxantrone [178]. As a single agent or in combination with bortezomib, GSK2606414 triggered apoptosis in multiple human myeloma cells [179]. Among the other described effects of GSK2606414 is synergistic efficacy with tumor-selective oncolytic reovirus type 3 Dearing in HNSCC HN5 and FaDu cells [180], an ability to reduce the volume of subcutaneous HN5 xenografts in mice treated with doxycycline and the reovirus [180], the suppression of the phenotypic transition of cancer-associated fibroblasts in pancreatic cancer [181], synergistic anti-tumor effects with digoxin on human leukemia cells K562 and THP-1 [182], the inhibition of PERK/eIF2α/CHOP pathway leading to accelerated apoptosis in small-cell lung cancer (SCLC) H1688 and H446 cells [183], and enhanced therapeutic responses of mice SCLC xenografts to the natural compound oridonin [183].

***GSK2656157*** is another highly selective ATP-competitive PERK blocker [184]. In pancreatic BxPC3 cells stressed by tunicamycin or TG, GSK2656157 suppressed PERK/eIF2 phosphorylation, decreased ATF4 and CHOP levels, and changed the expression of UPR-associated genes, while its oral administration inhibited the growth of pancreatic and multiple myeloma xenografts in mice accompanied by impaired tumor angiogenesis [185]. The growth of esophageal squamous cell carcinoma cells was also sensitive to GSK2656157 application [186]. However, an addition of GSK2656157 did not sensitize mice with subcutaneous leukemic K562 cell xenografts to imatinib [187]. Additionally, despite its anti-tumor activity, GSK2656157 exerts a serious off-tumor side effects associated with the inhibition of PERK activity in the pancreas, which leads to impaired pancreatic function [185].

***Other PERK inhibitors***. In the search for less toxic PERK inhibitors, a few other agents have been examined. The compound NCI 159,456 decreased the viability and increased the death of normal and ER-stressed non-small cell lung cancer A549 cells, which was accompanied by excessive ROS production, DNA damage, and increased mRNA levels of pro-apoptotic genes [188]. Cancer-associated PERK activity in rhabdomyosarcoma cell lines was diminished by the new PERK inhibitor AMGEN44 [189].

### 5.2. eIF2α Inhibitors

Since eIF2α phosphorylation can have pro-survival or apoptotic outcomes, both the suppression and stimulation of its activity have been proposed as potential strategies in anti-cancer therapy.

***ISRIB***. Integrated stress response inhibitor (ISRIB) is a small synthetic drug-like molecule that stabilizes eIF2B by binding its subunits, thus reversing eIF2α phosphorylation [190]. In spite of its insolubility, it showed a good safety profile and promising results in in vitro experiments and preclinical animal tumor models. ISRIB effectively decreased the growth and metastases of xenografts originating from patient-derived aggressive metastatic prostate cancer [64], lung tumor [191], and pancreatic duct adenocarcinoma SW 1990 cells with high level of BZW1 in mice [175], thus increasing the overall animal survival. It suppressed the migration and invasion of triple-negative breast cancer Hs576T and MDA-MB-231 cells overexpressing ETHE1 (ethylmalonic encephalopathy protein 1), which upregulates eIF2α phosphorylation [192], and diminished the growth and metastatic potential of subcutaneous MDA-MB-231 xenografts in mice either as a single agent or in combination with doxorubicin [193]. ISRIB and imatinib suppressed the proliferation of TG-stressed leukemic K562 and LAMA84 cells, whereas the administration of ISRIB to mice bearing K562 xenografts enhanced the sensitivity of the tumors to imatinib [187]. Unfortunately, in spite of its scientific value, the therapeutic potential of ISRIB in clinical trials, as well as its effectiveness, safety, and side effects, were not evaluated consistently [194].

***Salubrinal***. Salubrinal is an example of synthetic phosphatase inhibitor that specifically blocks eIF2α dephosphorylation by the complexes GADD34/PP1 and CREP/PP1, thus inducing sustained eIF2α hyperphosphorylation and eventually cell death [195,196]. It suppressed the proliferation of inflammatory breast cancer SUM149PT and SUM190PT cells by enhancing ROS production and changing the expression of ERS genes/proteins [197]. In adrenocortical carcinoma SW-13 and NCI-H295R cells, salubrinal increased the cytosolic Ca^2+^ content and upregulated components of the PERK/eIF2α/ATF4 and apoptosis signaling pathways [198]. A combination of salubrinal and ionizing radiation was proposed as a novel approach for the treatment of pediatric glioblastoma [199]. By disrupting the cell cycle and dephosphorylating cyclin A and cyclin-dependent kinases, salubrinal impaired the viability and clonogenic capacity of patient-derived and cultured (SCC4 and FaDu) head and neck squamous cells carcinomas, while in FaDu cells, it exerted synergistic or additive effects with chemotherapeutic drugs [200]. However, it did not enhance the sensitivity of glioblastoma U87 and U251 cells to the combination of temozolomide and simvastatin, although it promoted apoptosis as a single agent [176]. Excessive ROS production and enhanced death via the upregulation of xCT (SLC7A11), an antiporter of cystine and glutamate, was observed in breast, gastric, and oral squamous cell carcinoma cells treated with salubrinal under conditions of glucose deprivation [201]. Although ineffective as a single treatment, salubrinal in combination with 4E1RCat, an inhibitor of cap-dependent translation, synergistically suppressed the viability of melanoma UACC 903 cells and the development of subcutaneous UACC 903 xenograft tumors [202]. In triple-negative breast cancer BT549, SUM159 and MCF-10A cells, salubrinal potentiated the cytotoxicity and apoptosis induced by silver nanoparticles, AgNPs [203].

### 5.3. IRE1α/XBP1 Inhibitors

IRE1α inhibitors are classified as kinase (ATP-binding) domain and RNase domain blockers. The kinase inhibitors in turn include ATP-competitive agents that are able to stimulate the RNase domain, leading to XBP1 mRNA splicing (type I), and the compounds that inactivate the RNase (type II) [8,204]. The examples of type I IRE1α kinase inhibitors are APY29 and sunitinib, and the type II IRE1α kinase blockers are KIRAs (kinase inhibiting RNase attenuators) [204,205,206]. IRE1α RNase inhibitors include MKC-3946, MKC8866, 4µ8c, STF-83010, toyocamycin, salicylaldehydes, and hydroxyl-aryl-aldehydes [207,208,209,210]. They suppress XBP1 splicing, resulting in unresolved ER stress.

***Sunitinib***. Sunitinib, a synthetic indolinone derivative, has been approved for the standard first-line therapy of metastatic renal cell carcinoma (RCC) [211], and was examined as a part of combination therapy for ovarian [212] and breast [213] cancers. However, sunitinib is a multi-kinase inhibitor that also targets, for example, VEGF and PDGF receptors. Thus, although sunitinib alone caused a similar level of apoptosis to gemcitabine in PDAC cells and a synergized chemotherapeutic effect on mice with orthotopic PDAC xenografts, it did not suppress XBP1 splicing, which indicates an absence of its direct effect on IRE1α activity [100]. Additionally, in some cases, it showed poor tolerance alone [213]. Other significant obstacles are the development of resistance and lower efficacy than that of other drugs. For instance, the combined application of lenvatinib + pembrolizumab as the first-line treatment for patients with advanced RCC had higher therapeutic effect versus sunitinib as a standard [214].

***KIRAs***. The blockage of XBP1 splicing with KIRA6 led to apoptosis or suppressed the proliferation of cultured mast cell leukemia [215]. A more selective compound, 18/KIRA8, reduced the viability of multiple myeloma and non-myeloma cancer cell lines, diminished the growth of subcutaneous or orthometastatic myeloma xenograft tumors in mice, and potentiated the efficacy of the cytotoxic agents bortezomib and lenalidomide [216]. It also reversed the sensitivity of pancreatic tumor to radiotherapy [217].

***MKC-3946***. MKC-3946 exerted modest cytotoxicity toward multiple myeloma cells under in vitro conditions, but significantly suppressed the growth of subcutaneous myeloma in mice by inhibiting XBP1 splicing and enhancing the apoptotic effects of the anti-cancer drugs bortezomib and 17-AAG [207]. The cytotoxicity of MKC-3946 toward acute myeloid leukemia cells was also associated with the blockade of XBP1 mRNA splicing [83]. In stable (U87MG and A172) and patient-derived glioblastoma cells, MKC-3946 reduced the viability and colony formation, and potentiated the effect of temozolomide, particularly in cells with a methylated O^6^-methylguanine-DNA methyl transferase gene promoter [76].

***MKC8866***. MKC8866 (ORIN1001) is the only IRE1α inhibitor that entered clinical trials as an agent enhancing the effects of chemical/targeted therapy of advanced solid tumors [218]. In experiments, MKC8866 suppressed the proliferation and colony formation of mast leukemia HMC-1.2 [215] and rhabdomyosarcoma [189] cells. In prostate cancer cells and mouse xenograft models, MKC8866 was effective alone and exhibited additive or synergistic effects with antiandrogens (abiraterone acetate and enzalutamide) and taxanes (cabazitaxel and paclitaxel) [81]. It also increased the responsiveness of a mouse model of prostate cancer to anti-PD-1 antibody therapy [71]. In a series of breast cancer cells, MKC8866 treatment reduced the production of the pro-inflammatory factors IL-6, IL-8, CXCL1, GM-CSF, and TGFβ2, and enhanced the effectiveness of paclitaxel both in vitro and in vivo [219]. It increased the sensitivity of mice with intracerebral glioblastoma GL261-Luc to combined treatment with irradiation and temozolomide [220], and exerted synergistic effect with the tyrosine kinase inhibitor nilotinib on acute lymphoblastic leukemia [221] and with AZD1775 (an inhibitor of the WEE1 G2 checkpoint kinase) in ovarian cancer cells [222]. However, MKC8866 did not alter the rate of temozolomide + simvastatin-induced death of glioblastoma U87 and U251 cells, although it reduced the autophagic flux triggered by these drugs and decreased cell viability as a single agent [176].

***4μ8C***. This compound significantly inhibited the colony-forming ability of aggressive luminal B breast cancer SUM52 cells [79], suppressed the proliferation and migration of hepatocellular carcinoma HepG2 or Huh7 cells in vitro, and reduced fibrotic diethylnitrosamine-induced HCC tumor and collagen deposition in mice [223]. In both HCC xenograft and in vitro HCC models (HepG2, SNU449, Huh7 cells, and patient-derived organoids), 4μ8C synergized with doxorubicin’s effects by altering lipid metabolism and the oxygen consumption rate, and downregulated ERS markers [224]. It was effective at suppressing the proliferation of colon cancer stem cells in vitro and enhanced the efficacy of 5-FU chemotherapy in mice bearing 5-FU-resistant colon cancer [225,226].

***STF-083010***. The IRE1α RNase-specific inhibitor, STF-083010, induced apoptosis and blocked the growth of mast cell leukemia [215], and increased miR-34a expression in a few AML cell lines [83]. It suppressed the proliferation of a series of pancreatic cancer cell lines by increasing the expression of apoptotic proteins and arresting the cell cycle at G1 or G2/M phases, and potentiated the effect of bortezomib [227]. In tunicamycin-stressed PDAC cells, STF-083010 increased the number of mature autophagy-associated lysosomes and had additive effect with gemcitabine [100]. It restored the sensitivity of breast cancer MCF7 cells to tamoxifen in vitro, while both drugs synergistically suppressed the growth of mammary tumors in mice [80]. However, although STF-083010 showed anti-tumor effects in experimental settings, it is unstable in organisms and its application in clinical trials is under question.

***Other IRE1α RNase inhibitors***. HNA (2-hydroxy-1-naphthaldehyde), toyocamycin (an adenosine analog produced by Actinomycetes), and 3ETH (3-ethoxy-5,6-dibromosalicylaldehyde) suppressed XBP1 mRNA splicing in acute myeloid leukemia cells, followed by caspase-dependent apoptosis, G1 cell cycle arrest, and altered expression of chaperones, Bcl-2 family, G1 phase-controlling proteins, and miR-34a, whereas its synergic effect with bortezomib was linked with p-JNK and p-PI3K [83]. The blockade of tunicamycin-induced XBP1 splicing in pancreatic cancer cells by these compounds reduced proliferation and the colony-forming ability, arrested the cell cycle at G1 or G2/M phases, enhanced the expression of apoptotic proteins, decreased the mitochondrial membrane potential, and potentiated the anti-tumor activity of bortezomib in mice with pancreatic cancer xenograft [227]. The urea-based compound Z4P was shown to diminish the migratory capacity of glioblastoma cells (U251, RADH85, and RADH87), arrested IRE1 phosphorylation in U87 cells, reduced the burden of xenograft tumors in mice, prolonged relapse-free survival, and sensitized glioblastoma to temozolomide [228].

### 5.4. ATF6 Inhibitors

The inhibitors of ATF6 signaling are rare. Two decades ago, a serine protease inhibitor AEBSF (4-(2-aminoethyl) benzenesulfonyl fluoride) was shown to prevent the cleavage of ATF6α/ATF6β, thus suppressing the transcription of ATF6 target genes [229]. However, the only highly specific inhibitors of ATF6α developed to date are the pyrazole amides ceapins [230]. Ceapin-A7 impaired the survival of castration-resistance prostate cancer (CRPC) cells [231] and sensitized the radioresistant pancreatic cancer cells to radiotherapy by increasing apoptosis and G1 cell cycle arrest [217].

### 5.5. GRP78 Inhibitors

***YUM70***. YUM70 is a newly discovered derivative of 8-hydroxyquinoline that inhibits GRP78 enzymatic activity by direct binding. Such ability impairs the potency of GRP78 to correct misfolded proteins and adapt to ERS. As a monotherapy, YUM70 activated ERS-associated apoptosis, suppressed the growth of pancreatic cancer cells in vitro, and reduced the subcutaneous pancreatic PaCa-2 tumors in mice without toxicity to other organs, while when administered in combination with the cytotoxic drugs topotecan, vorinostat, MG132, and 5-FU, it had synergic or additive effects on the clonogenic capacity of cells [232]. It efficiently inhibited oncogenic KRAS expression in a panel of human lung, colon, and pancreatic cancer cell lines both in vitro and in vivo [233]. The addition of YUM70 reversed the sensitivity of cisplatin-resistant HNSCC to chemotherapy by reducing their viability and clonogenic capacity [234]. The treatment of HNSCC (SCC25 and SCC15) cells, their cisplatin-resistant clones, TNBC (MDA-MB-231), and PDAC (MIA PaCa-2) cells with YUM70 diminished the expression of oncogenic protein c-Myc, which led to apoptosis, and suppressed the growth of pancreatic xenografts [235].

***HA15***. Another potent and specific GRP78 inhibitor is thiazole benzenesulfonamide HA15, which suppresses its ATPase activity by promoting conformational changes. It triggered early ERS followed by apoptosis and autophagy in a series of melanoma cell lines, including patient-derived melanocytes resistant to BRAF inhibitors, and suppressed the growth of BRAF-resistant melanoma xenografts in mice [236]. In adrenocortical carcinoma H295R cells, HA15 suppressed proliferation and steroidogenesis [237]. Its ability to reduce the stemness led to the apoptosis of colorectal cancer HCT116 and HT29 cells and impaired formation of tumor spheres [238], while in a few lung, colon, and pancreatic cancer cell lines, it diminished oncogenic KRAS expression [233]. HA15-induced apoptosis in lung cancer cells was accompanied by the formation of autophagosomes with increased expression of apoptosis and autophagy genes [101]. The pro-apoptotic effects of HA15 in NSCLC HCC827-GR, H1993-GR and H1993-ER cells with acquired resistance to therapeutic EGFR tyrosine kinase inhibitors included enhanced ROS production [239]. Similar to YUM70, HA15 reduced c-Myc expression and upregulated the eukaryotic translation inhibitor 4E-BP1 in multiple c-Myc-dependent 2D cell culture, 3D spheroid, and xenograft cancer models [235].

***Epigallocatechin gallate***. The compound (−)-Epigallocatechin-3-gallate (EGCG) is one of the polyphenol flavonoids found in green tea. It is believed to directly interact with GRP78 at its ATP-binding site, thus inducing conformational changes and preventing the formation of the anti-apoptotic GRP78/caspase-7 complex [240]. EGCG has been intensively studied as a part of preventive therapy for various types of malignancies, including gliomas [98,241], breast [240,242], adrenal [243], and colorectal [244] cancers. Although poorly effective as a single agent, it sensitized breast cancer cells to etoposide [240] and paclitaxel in vitro and in vivo by enhancing JNK phosphorylation [242]. The increased sensitivity of glioma cells to temozolomide in the presence of EGCG included the activation of CHOP and caspase-7 [98]. Although EGCG did not improve the survival of mice with intracranially implanted U87 (p53 wild type) or U251 (p53 mutant) glioblastoma cells, it significantly enhanced the effect of temozolomide in combination therapy [245]. However, the cellular effects of EGCG are variable and cannot be attributed solely to GRP78 suppression.

**Table 4:** Examples of using inhibitors of UPR effectors in experimental cancer treatment.

Cell Type	Treatment	Molecular Changes	Cellular Effects	References
		**PERK inhibitors**		
PDAC SW1990 cells with ↑ BZW1	GSK2606414 10 µM 12 h	↓ p-eIF2α, ↓ HIF1α, ↓ c-Myc, ↓ HIF1A, ↓ MYC IRES	↓ Cell survival ↓ Proliferation	[175]
Mice with PDAC SW1990 BZW1 xenografts	GSK2606414 100 mg/kg IP twice/w	↓ Ki67 staining ↓ TUNEL staining	↓ Tumor growth ↓ Cell proliferation ↑ Apoptosis	[175]
Glioblastoma U87 and U251 cells	GSK2606414 1–20 µM + simvastatin + TMZ 72 h	↑ p62, ↓ p-eIF2α, ↓ LC3B-II/I in U87 cells, ↑ LC3B-II/I in U251 cells	↓ Viability ↑ Sensitivity to simvastatin+TMZ	[176]
Multidrug-resistant colorectal cancer S1-M1–80 cells	GSK2606414 1–3 µM + mitoxantrone or DOX 10 µM 24–72 h		↑ Sensitivity to mitoxantrone and DOX	[178]
Human myeloma L363, H929, U266, and KMS11 cells	GSK2606414 1–100 µM or 10 µM + BTZ 4 nM 24–48 h	↓ PERK, ↓ ATF4, ↓ eIF2α, Changes in the expression of UPR genes	↓ Cell survival ↑ Apoptosis ↑ Sensitivity to BTZ	[179]
SCLC H1688 and H446 cells	GSK2606414 10 µM + Oridonin 20 µM 24 h	↓ p62 ↓ LC3B-II/LC3B-I	↑ Apoptosis, ↑ Oridonin effect, ↑ Autophagy	[183]
Mice with SCLC H1688 cell xenografts	GSK2606414 50 mg/kg + oridonin 10 mg/kg	↓ GRP78, ↓ p-PERK, ↓ p-eIF2α, ↓ ATF4, ↓ CHOP	↓ Tumor size	[183]
Himan pancreatic adenocarcinoma BxPC3 cells	GSK2656157 1 µM + tunicamycin or TG 6 h	↓ p-PERK, ↓ ATF4, ↓ p-eIF2α, ↓ CHOP, ↓ UPR gene expression		[185]
Mice with pancreatic cancer xenografts	GSK2656157 50 or 150 mg/kg twice/d OR 14 d	↓ p-PERK, changes in genes expression	↓ Tumor growth ↓ Blood vessel density	[185]
Myeloid leukemia K562 and LAMA 84 cells	GSK2656157 0.1–10 µM + TG 100 nM + IMA 1 μM 16 h	↓ CHOP mRNA ↓ GADD34 mRNA		[187]
Mice subcutaneous K562 xenograft	GSK2656157 20 mg/kg/d + IMA 50 mg/kg twice/d 2 w		No significant decrease in the tumor mass	[187]
Intact and ER-stressed NSCLC A549 lines	NCI 159456 3–100 or 50 µM + TG 500 nM 24 h	DNA damage, ↑ *ATF4*, ↑ *DDTI3*, ↑ *BAX*, ↓ *BCL2*, ↑ Caspase-3, ↑ ROS	↓ Viability ↑ Apoptosis	[188]
**eIF2 inhibitors**				
Mice with prostate cancer xenografts	ISRIB 10 mg/kg, 6 w		↓ Tumor growth, ↓ Metastases, ↑ Survival	[64]
Mice with PDAC SW1990 BZW1 cell xenografts	ISRIB 2.5 mg/kg IP twice/w		↓ Tumor volume ↑ Animal survival	[175]
TNBC Hs576T and MDA-MB-231/ETHE1 cells	ISRIB 200 nM 24 h	↓ p-eIF2 ↓ ATF4	↓ Migration ↓ Invasion	[192]
Mice with subcutaneous TNBC MDA-MB-231 cell xenografts	ISRIB for 15 d + DOX 6 injections	↑ Cleaved caspase-3	↓ Tumor volume ↓ Tumor weight ↑ Sensitivity to DOX	[193]
Human ML K562 and LAMA84 cells	ISRIB 250 nM + IMA 0.5–1 µM 16 h	↓ p-STAT, ↓ m-TOR, ↓ p-GSK3	↓ Proliferation	[187]
Mice with subcutaneous K562 xenografts	ISRIB 2 mg/kg/d + IMA 100 mg/kg/d 2 w		↑ Sensitivity to IMA ↓ Tumor mass	[187]
Inflammatory breast cancer SUM149PT and SUM190PT cells	Salubrinal 10 µM 24–48 h	↑ p-eIF2a, ↓ PERK, ↑ CHOP, ↓ GRP78, ↑ ATF4, ↑ ROS, ↑ Bax, ↑ cleaved PARP, ↑ Caspase-3, ↓ p-Akt, ↓ p-NFkB	↓ Proliferation	[197]
ACC SW-13 and NCI–H295R cells	Salubrinal 100 µM 24 h	↑ p-eIF2α, ↑ p-PERK, ↑ ATF4, ↑ Ca^2+^, ↑Bcl-2	↓ Viability, migration ↑ Apoptosis	[198]
Primary pediatric GB SU-DIPG and KNS-42 lines	Salubrinal 2.5–8 µM + irradiation	↑ p-eIF2a	↑ Sensitivity to irradiation ↓ Cell survival	[199]
HNSCC SCC4 and FaDu cells, patient-derived 3D spheres	Salubrinal 10–50 µM 24–72 h	↑ p-eIF2a, ↓ p-RB1, ↓ E2F1, ↓ Cyclin A, ↑ p21	↓ Viability ↓ Clonogenic ability Cell cycle arrest	[200]
Glioblastoma U87 and U251 cells	Salubrinal 1–20 µM or 15 µM + simvastatin + temozolomide 72 h	↑ p-eIF2α	↓ Viability No synergistic effect with cytotoxic drugs	[176]
Melanoma UACC 903 cells	Salubrinal 40 µM + 4E1RCat 10 µM 48 h	↓ Protein synthesis, ↓ Cyclins, ↓ CDK2, ↓ Polysomes	↓ Cell cycle progression ↓ Viability	[202]
Mice with subcutaneous UACC 903 melanoma	Salubrinal 1 mg/kg + 4E1RCat 2.5–15 mg/kg I P one/2 d 3–4 w		↓Tumor volume	[202]
TNBS BT549, SUM159, and MCF-10A cells	Salubrinal 5–10 µM + AgNPs 24 h	↑ p-eIF2a, ↑ CHOP, ↑ cleaved Caspases-3/7/9	↓ Viability ↑ AgN-induced death	[203]
**IRE1 inhibitors**				
PDAC cells Panc3.27, Pan02, Miapaca-2	Sunitinib 10 µM + GEM 100–250 nM 48–72 h	↓ Lysosomal degradation, DNA fragmentation	↓ Viability, ↑ Apoptosis, ↓ Autophagy, ↑ GEM effect	[100]
Mice with orthotopic PDAC Pan02 or KPCP1 xenografts	Sunitinib 25 mg/kg/d OR + GEM 25 mg/kg/w IP + PTX 10 mg/kg/w IP until mortality or for 4 w	↓ Ki67-positive cells ↓ TUNEL-positive cells ↓ GRP78 immunosignal	↓ Tumor growth ↑ Overall survival ↑ Chemotherapy effect	[100]
Myeloma patient-derived INA6 and RPMI 8226 cells	MKC-3946 10 µM + BTZ 2.5–10 nM or 17-AAG 125–1000 nM 2–24 h	↓ XBP1s, ↑ CHOP, ↑ ATF4, ↑ p-eIF2α, ↑ Caspase-3, ↑ cleaved PARP	↑ Growth inhibition ↑ Apoptosis ↑ BTZ, 17-AAG effect	[207]
Mice with subcutaneous RPMI 8226 myeloma	MKC-3946 100 mg/kg/d IP + BTZ 0.15 mg/kg IV 2/w 21 d	↓ XBP1s ↑ CHOP mRNA	↓ Tumor growth ↑ Overall survival	[207]
GBM patient-derived U87MG, A172, BAH1 TMZ-resistant cells	MKC-3496 10 μM + TMZ 50 μM 24–72 h	↓ sXBP1 mRNA	↓ Colony formation ↓ Viability ↑ Efficacy of TMZ	[76]
Mouse prostate cancer LNCaP, VCap, 22Rv1, and C4–2B cells xenograft models	MKC8866 300 mg/kg/d or once/2 d OR + enzalutamide, abiraterone acetate, cabazitaxel, PTX	↓ sXBP1 ↑ Cleaved Caspase-3 ↓ PCNA	↓ Tumor growth ↓ Proliferation, Additive/synergic effects with anti-cancer drugs, ↑ apoptosis	[81]
Mice with subcutaneous Myc-CaP prostate cancer xenografts	MKC8866 150–300 mg/kg/2 d + anti-PD-1 10 mg/kg/w IP 18–38 d		↓ Tumor volume/weight ↑ Anti-PD-1 immunotherapy	[71]
Breast cancer cells MCF7, SKBR3, MDA-MB-231	MKC8866 5–20 μM + PTX 10 nM 72 h	↓ XBP1s, ↓ IL-6, ↓ IL-8, ↓ CXCL1, ↓ TGFβ	↓ Proliferation Cell cycle arrest ↓ Mammospheres	[219]
Mice with MDA-MB-231 xenografts	MKC8866 300 mg/kg/d OR + PTX 10 mg/kg/w IV up to 60 d	↓ XBP1s	↓ Tumor growth, ↑ sensitivity to PTX ↑ Survival	[219]
Glioblastoma U87 and U251 cells	MKC8866 10–80 µM or 30 µM + SIM and TMZ 72 h	↓ p62, ↓ Beclin-1, ↓ XBP1s, ↑ LC3B-II/LC3B-I in U251 cells	↓ Viability No synergistic effect with TMZ and SIM on death	[176]
Mice with intracerebral GL261-Luc cell glioblastoma	MKC8866 + IR 2 Gy + TMZ 25 mg/kg 5 d → TMZ 30–50 mg/kg 4 w	↑ Active caspase-3	↑ Survival ↑ Apoptosis	[220]
HCC HepG2, Huh7 + stellate LX2 cells	4µ8C 50–100 µM 48 h	↓ PCNA mRNA ↓ ROS	↓ Proliferation ↓ Migration	[223]
Mice with DEN-induced HCC	4µ8C 10 mg/g 2/w until 25th week	↓ Oncogenic proteins ↓ PCNA, ↓ HCC promoters PRDX5 and DDAH1, ↓ sXBP1/XBP1	↓ Tumor growth ↓ Collagen deposition ↓ Smooth muscle actin	[223]
HCC cells (HepG2, SNU449, Huh7) and patient-derived organoids	4µ8C 10–1000 µM + DOX 1 µM 24 h	↓ sXBP1 ↓ ATF4 ↓ Lipid metabolism genes expression	↓ Viability, ↑ Death, ↓ lipid metabolism, ↑ DOX effect, ↓ oxygen consumption	[224]
Mice HCC tumor induced by DEN	4µ8C 10 mg/g IP + DOX 4 mg/g IV bi-weekly 3 w	↑ Caspase-3, ↓ ATF4, ↓ Ki67-positive cells, ↓ aSMA mRNA, ↓ CHOP, ↓ CXCL4, ↓ IL-1, ↓ ALT,	↓ Tumors, ↑ DOX effect, ↓ CD68, ↓ Triglycerides, ↓ Inflammation, ↓ Fibrosis, ↓ Collagen	[224]
Patient-derived and blast AML cells	STF-083010 50 μM 24 h	↓ sXBP1 ↑ miR-34a expression	↑ Cytotoxicity	[83]
Mice with breast cancer MCF-7-TAM-resistant xenografts	STF-083010 30 mg/kg/w + TAM 100 µg/kg/d IP	↓ sXBP1	↑ Effect of TAM ↓ Tumor growth ↑ Caspase-3	[80]
Patient-derived AML blast cells	HNA 2–25 μM + BTZ 2.5–10 μM 48–72 h	↓ sXBP1, ↑ CHOP, ↓ Bcl-2, ↑ Bim, ↓ Cyclin D, ↑ p21cip1, ↑ p27kip1, ↑ Cleaved PARP, ↑ Caspase-3, ↑ miR-34a	↑ Cytotoxicity, ↑ BTZ effect, ↓ Colony formation, ↓ Viability, ↑ Apoptosis	[83]
Pancreatic cancer cells MiaPaCa-2, SU8686, Panc0403, and Panc0327	HNA 10–50 μM 6–24 h	↓ sXBP1, ↑ CHOP, ↑ p-JNK, ↑ cells in sub-G1 phase, ↑ cleaved PARP, ↓ Bcl-2, ↑ Bim	↑ Apoptosis, ↓ Colony formation, ↓ Proliferation, ↓ MMP	[227]
Pancreatic cancer cells MiaPaCa-2, SU8686, Panc0403, and Panc0327	Toyocamycin 0.5–5 μM 24 h	↑ Cleaved PARP ↓ Bcl-2 ↑ CHOP	↓ Proliferation ↓ Colony formation ↓ Mitochondrial membrane potential	[227]
Pancreatic cancer cells MiaPaCa-2, SU8686, Panc0403, and Panc0327	3ETH 1–10 μM 24 h		↓ Proliferation ↓ Colony formation	[227]
Mice with pancreatic BxPc3 xenografts	3ETH 20 mg/kg 3 times/w 4 w		↓ Tumor growth	[227]
Glioblastoma U87 cells	Z4 0.5–25 μM 4–24 h	↓ sXBP1, ↓ p-IRE1, ↓ SPARC	↓ Viability, ↓ Migration	[228]
Mice with orthotopic glioblastoma	Z4 300 mg/kg/d 182 d +TMZ 10 mg/kg/d 21d		↑ Effect of TMZ ↑ Relapse-free survival	[228]
**GRP78 inhibitors**				
Pancreatic cancer PaCa-2, PANC, and BxPC-3 cells	YUM70 1–5–15 µM 24–48 h	↑ GRP78, ↑ CHOP, ↑ FAM 129A, ↑ p-eIF2α, ↑ ATF4, ↓ c-MYC, ↓ eIF4A, eIF4E, ↓ eIF5A, ↑ 4E-BP1, ↓ p-4E-BP1, ↑ cleaved PARP, ↑ Caspase-3/7	↓ Viability, ↑ Apoptosis, ↓ Cell proliferation, ↓ Colony formation, Synergistic/additive effects with topotecan, vorinostat, or 5-FU	[232]
Mice bearing PaCa-2 cell xenografts	YUM70 30 mg/kg IP 5 d/w 7 w	↓ Ki67 staining, ↑ CHOP, ↑ FAM 129A, ↑ Caspase-3	↓ Tumor growth ↑ Apoptosis	[233,234]
HNSCC cells SCC15, SCC25, and SCC351	YUM70 1.25–30 µM + CIS 12–24 µM 48 h	↑ GRP78, ↑ CHOP, ↑ cleaved PARP, ↑ Caspase-7	↓ Viability, ↑ Apoptosis, ↓ Clonogenicity, ↑ Sensitivity to CIS	[235,236]
TNBC MDA-MB-231 cells and HNSCC SCC15 and SCC25 cells	YUM70 10 μM 24 h	↓ c-MYC, ↓ eIF4A, ↓ eIF4E, ↓ eIF5A, ↑ 4E-BP1, ↓ p-4E-BP1, ↑ cleaved PARP	↓ Viability ↑ Apoptosis	[235]
Melanoma A375, Mel501, SKMel28, and patient-derived cells	HA15 10 μM, 48 h or 1–24 h	↑ p-PERK/*EIF2AK3*, ↑ p-elF2a, ↑ *ATF4*, ↑ *ERN1*, ↑ *ATF6*, ↑ *DDIT3*, ↑ *LC3*, ↑ *sXBP1*, ↑ *JUN*, ↑ *BCL2*	↓ Viability ↑ ER stress ↑ Apoptosis ↑ Autophagy	[236]
Mice with melanoma A375 xenografts sensitive/resistant to BRAF inhibitors	HA15 0.7 mg/day 2 w	↑ CHOP ↑ LC3B ↑ Autophagosomes	↓ Tumor growth ↑ Apoptosis ↑ Autophagy	[236]
Lung cancer A549, H460, and H1975 cells	HA15 2–10 μM 48 h or 10 μM 24 h	↑ *ATF4*, ↑ *ATF6*, ↑ *XBP1*, ↑ *IRE1*, ↑ *CHOP*, ↑ *Atg5*, ↑ *Atg7*, ↑ *Atg12*, ↑ *LC3*, ↑ *ULK1*, ↑ Bax, ↑ CHOP	↓ Proliferation, Cell cycle arrest, ↑ Autophagosomes, ↓ Viability, ↑ Apoptosis	[239]
Pancreatic, lung, and colon cancer cells, KRAS mutant	HA15 10 μM 2 4–48 h	↑ Cleaved PARP, ↑ CHOP, ↑ Caspase-7	↓ Viability ↑ Apoptosis	[233]
HNSSCC SCC25 and SCC15 cells, TNBC MDA-MB-231 cells	HA15 10 μM 24–48 h	↓ c-MYC, ↑ 4E-BP1, ↓ p-4E-BP1, ↓ eIF4A, ↓ eIF4E, ↓ eIF5A	↓ Viability ↑ Apoptosis	[101]
Breast cancer MDA-MB-231 and T-47D cells	EGCG 10 μM + ETO 20–40 μM 24-48 h	↑ Caspase-7, ↓ GRP78/caspase-7 complex	↑ Apoptosis ↓ Colony formation ↑ Sensitivity to ETO	[241]
Breast cancer cells 4T1, MCF-7, and MDA-MB-231	EGCG 20 μM + PTX 1 μM 48 h	↑ p-JNK ↓ GRP78	↑ Apoptosis ↑ Sensitivity to PTX	[242]
Mice with breast 4T1 xenografts	EGCG 30 mg/kg + PTX 10 mg/kg IP 24 d	↑ p-JNK ↓ GRP78	↓ Tumor growth	[242]

Abbreviations. PDAC—pancreatic duct adenocarcinoma, HCC—hepatocellular carcinoma, MM—multiple myeloma, GBM—glioblastoma, HHC—hepatocellular carcinoma, AML—acute myeloid leukemia, TNBC—triple-negative breast cancer, HNSCC—head and neck squamous cell carcinoma, NSCLC—non-small cell lung carcinoma, OSCC—oral squamous cell carcinoma, TMZ—temozolomide, BTZ—bortezomib, PTX—paclitaxel, GEM—gemcitabine, CIS—cisplatin, IMA—imatinib, SIM—simvastatin, TM—tamoxifen, ETO—etoposide, DEN—diethylnitrosamine, HNA—2-hydroxy-1-naphthaldehyde, 3ETH—3-ethoxy-5,6-dibromosalicylaldehyde, IR—irradiation, PCNA—proliferating cell nuclear antigen, MMP—mitochondrial membrane potential, ↑—increase, ↓—suppression.

## 6. Conclusions

Numerous experimental in vitro and in vivo studies have shown that enhanced ERS and abnormal activities of components of the protein degradation machinery are common phenomena that are implicated in tumor growth, metastasis, and the emergence of resistance to chemotherapy. Modulation of ERS and the UPR is believed to be a promising and valuable approach to sensitize the malignancies of different origins to conventional cytotoxic drugs. The attempts to overcome the adaptation of cancer cells to apoptosis resulted in development of a variety of natural and synthetic compounds that are able to selectively suppress or activate ERS sensors and the components of UPR downstream signaling pathways. However, the majority of agents targeting ERS/UPR effectors have been examined only in experimental in vitro and in vivo settings. Although many of these compounds have entered clinical trials (clinicaltrials.gov database), only few of them, like sunitinib, have been approved for routine anti-cancer therapy. Moreover, the number of completed clinical trials for which the results have been published in scientific journals and detailed descriptions of the tumor types, number of patients, side effects and proper statistical analysis have been provided is limited. As a result, the available data are heterogeneous, inconsistent and often contradictory. The possible cause of such a situation is the quite variable expression of ERS/UPR genes and proteins in different cancers, which greatly depends on the cell type and presence of mutations. It means that the application of the agents targeting ERS/UPR components cannot be universal. The existence of other cellular targets, possible toxicities to normal cells, drug–drug interactions, poor bioavailability, insolubility, and other factors are still obstacles. The design of more specific compounds with low toxicity, the absence of side effects, and better bioavailability, either as the derivatives of existing natural drugs or new synthetic agents, is urgently required. Another way to increase the therapeutic specificity of ERS/UPR-modulating compounds is the development of more efficient delivery systems like nanoparticles and liposomes.

## Figures and Tables

**Table 1:** Examples of aberrant SERCA expression in human cancer tissues.

Scheme	Expression Profile	Cancer Type	Clinical Outcome	References
**SERCA1**	Overexpression	Breast cancer	Poor prognosis Reduced survival	[30]
		Colorectal carcinoma		[31]
**SERCA2**	Overexpression	Colon and rectal adenomas and carcinomas	Lower survival Invasion Metastasis	[32,33,34]
**SERCA3**	Downregulation	Gastric carcinoma	Metastasis Poor prognosis	[35,36]
		Glioma/glioblastoma		[37]
		Colorectal carcinoma	No correlation with survival	[38]
		Choroid plexus papillomas and carcinomas	-	[39]
		Breast carcinomas		[41]

Comments: - not analyzed.

## Data Availability

Not applicable.
